# Continuous Polyol Synthesis of Metal and Metal Oxide Nanoparticles Using a Segmented Flow Tubular Reactor (SFTR)

**DOI:** 10.3390/molecules200610566

**Published:** 2015-06-08

**Authors:** Andrea Testino, Frank Pilger, Mattia Alberto Lucchini, Jose Enrico Q. Quinsaat, Christoph Stähli, Paul Bowen

**Affiliations:** 1 Paul Scherrer Institut, Villigen PSI, CH-5232 Switzerland; E-Mails: frank.pilger@psi.ch (F.P.); mattia.lucchini@mat.ethz.ch (M.A.L.); 2 Department of Materials, ETH Zürich, Vladimir-Prelog-Weg 5, CH-8093 Zurich, Switzerland; 3 EMPA, Laboratory for Functional Polymers, Überlandstrasse 129, CH-8600 Dübendorf, Switzerland; E-Mail: Jose-Enrico.Quinsaat@empa.ch; 4 Powder Technology Laboratory (LTP), EPFL, MXD 336 CH-1015 Lausanne, Switzerland; E-Mail: paul.bowen@epfl.ch; 5 RMS Foundation, Bischmattstrasse 12, CH-2544 Bettlach, Switzerland; E-Mail: christoph.staehli@rms-foundation.ch

**Keywords:** segmented flow tubular reactor, SFTR, continuous polyol synthesis, continuous nanoparticle synthesis, ethylene glycol

## Abstract

Over the last years a new type of tubular plug flow reactor, the segmented flow tubular reactor (SFTR), has proven its versatility and robustness through the water-based synthesis of precipitates as varied as CaCO_3_, BaTiO_3_, Mn_(1−x)_Ni_x_C_2_O_4_·2H_2_O, YBa oxalates, copper oxalate, ZnS, ZnO, iron oxides, and TiO_2_ produced with a high powder quality (phase composition, particle size, and shape) and high reproducibility. The SFTR has been developed to overcome the classical problems of powder production scale-up from batch processes, which are mainly linked with mass and heat transfer. Recently, the SFTR concept has been further developed and applied for the synthesis of metals, metal oxides, and salts in form of nano- or micro-particles in organic solvents. This has been done by increasing the working temperature and modifying the particle carrying solvent. In this paper we summarize the experimental results for four materials prepared according to the polyol synthesis route combined with the SFTR. CeO_2_, Ni, Ag, and Ca_3_(PO_4_)_2_ nanoparticles (NPs) can be obtained with a production rate of about 1–10 g per h. The production was carried out for several hours with constant product quality. These findings further corroborate the reliability and versatility of the SFTR for high throughput powder production.

## 1. Introduction

The SFTR concept was revealed at the École Polytechnique Fédérale de Lausanne (EPFL) on 15 July 1996 when the invention was disclosed in a patent application [1]. The main motivation was the development of a new technology devoted to overcome the main limitations associated to the mass and heat transport issues during the scale up of high-tech laboratory chemical production in the form of sub-micrometric powders. In fact, although many excellent powders have been discovered and prepared in laboratories at the mg level, transferring these processes to the kg production scale is often a bottleneck that hinders the creation of new innovative materials.

In order to prepare materials with well-defined particle size, morphology, stoichiometry and polymorphic phase, it is of paramount importance to maintain control over the elementary processes which govern particle formation, namely nucleation, growth, and agglomeration. These elementary processes are strongly influenced by the local conditions that each particle sees in its local environment; every small temperature or chemical speciation variations around the growing particle defines new conditions driving each particle towards different morphology, size, or phase. It is a matter of fact that increasing the batch size, for instance from a few milliliters to several liters, the precise local control over the transport processes is strongly compromised. As a consequence, the ideal properties of products obtained in small batches disappear upon upscaling. Thus, the option to intensify the production by simply increasing the batch size while maintaining the quality is not realizable: the volume of the reactor has to be maintained comparably small. The innovative step introduced with the SFTR concept is able to cope with the upscaling problem: instead of increasing the batch size, the number of small reactors is multiplied. In the SFTR the physicochemical transformation occurs in droplets (the small reactors) confined in a tubular reactor and segmented by a secondary fluid. The secondary fluid (the segmenting fluid, e.g., *n*-dodecane) has to be immiscible with the fluid (in this paper ethylene glycol, EG) where the precipitation occurs.

The segmentation is a key feature of the concept since it completely hinders the axial back mixing, typical for non-segmented tubular reactors, enabling dynamic ideal plug-flow and hinders the possibility of the reactant mixture to come in contact with the reactor wall. In fact, the fluid used to separate (segment) the droplets wet the inner wall of the tubular reactor forming a protective layer [2]. Thus, the solid matter in formation cannot nucleate nor grow on the tubular wall (known as ‘fouling’) allowing the continuous production for several hours (>12 h) with constant product quality.

**Figure 1:**
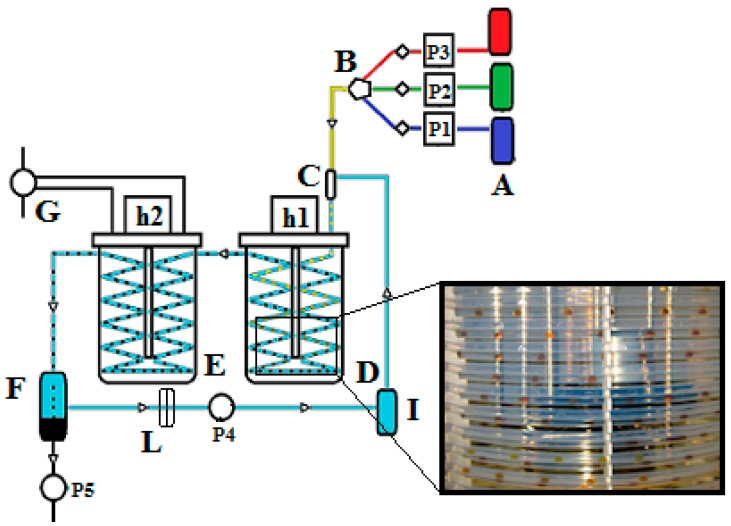
Sketch of the SFTR reactor. A: Pumping system composed by three HPLC pumps (P1, P2, P3) pulsation dumpers and back pressure valves; B: Mixer; C: Segmenter; D: Thermostatic bath equipped with heating head (h1) and mechanical stirrer; E: cooling bath equipped with heating head (h2) and mechanical stirrer and connected to the chiller (G); F: decanter connected to the pump P5; I: reservoir; L: filter; P4: circulating pump.

A schematic view of the SFTR is given in Figure 1. The SFTR is composed of three distinct parts: (i) a micromixer which ensures that the reactants are efficiently mixed; (ii) a segmenter; and (iii) a tubular reactor, placed in a thermostatic bath. 

The droplets (about 0.2 cm^3^) circulate through the tube, each with an identical *history* (e.g., residence time and heat exchange). The precipitation starts in the mixing chamber and/or is induced by the rapid temperature change when the droplets are heated in the thermostatic bath. According to fluid dynamic modeling, the temperature of the droplets reaches 95% and 99% of the bath temperature within 6 and 10 s, respectively [3]. 

The residence time in the tubular reactor is determined by the pump flow rates and the tube length and can be adjusted according to the specific reaction kinetics. If needed, a heat-exchanger can be placed after the tubular reactor in the process line to cool down the reacting mixture and quench the reaction. Finally, a separation unit (or decanter) allows the separation of the product and the immiscible fluid; the product—particle in suspension—is collected, while the immiscible fluid is recycled in the process for environmental and cost concerns. 

As a result of the control of the entire precipitation pathway, particle size distribution width, phase purity, and morphology are significantly improved even comparing the SFTR products with those produced in few milliliter size batch reactors [4,5,6,7].

To increase the throughput for commercial applications, the SFTR can be “scaled-out” by multiplying the number of tubes running in parallel. The production of calcium carbonate with a scaling-out factor of 5000 with no change in product quality and the robustness of the process in the production of high quality ultrafine barium titanate powders were already demonstrated [4].

The concept of keeping the scale rather small in order to increase the process control was introduced in the 70’s with the first microfluidic devices; twenty years later “microfluidics” became a hot topic. The first report where the concepts of “microfluidic” and “segmented flow” were combined was published in 2003 and applied for cell cultivation [8]. Microfluidics deal with tiny amounts of liquids (microliters) confined in microchannels. This technology certainly allows an excellent control over heat and mass transport; on the other hand it is not an option for any industrial production. To a certain extent, the segmented microfluidic concept is the scale-down of the SFTR reactor, at least three orders of magnitude smaller, not applicable for material production, and presented 7 years later.

SFTR competitive technologies are becoming commercially available, e.g., Accendo Corporation segmented flow chemistry [9], and Orbis droplet technologies [10]. They have some common concept points but do not have the technological flexibility. In particular, these technologies are suitable for chemical synthesis but they are not able to handle particulate fluids, *i.e.*, they are not suitable for nanoparticle synthesis.

SFTR prototypes specifically developed for water based powder production are nowadays available in several European laboratories. In the recent years, the SFTR concept has been further extended to work with organic solvents. In this paper we present the latest results on nanopowders synthesis prepared with the SFTR and in ethylene glycol (EG). Thanks to the high boiling temperature of this organic solvent, the syntheses can be carried out up to 180 °C, mimicking the solvothermal conditions applied in autoclaves but with the aforementioned advantages. Four materials will be discussed: two metal NPs (Ag and Ni), a metal oxide (CeO_2_) and a salt (β-tricalcium phosphate, Ca_3_(PO_4_)_2_).

## 2. Results and Discussion

### 2.1. SFTR Polyol Synthesis of CeO_2_ NPs

CeO_2_ has received much success in redox/combustion catalysts due to its ability to shift between reduced and oxidized states as a result of changes in gas phase oxygen concentration. As an oxygen storage component, ceria acts as an oxygen buffer for optimal conversion in automotive three-way catalyst (TWC) systems, providing oxygen under lean conditions and removing it under rich conditions. Ceria’s superior oxygen storage capacity (OSC) is due to the underlying redox equilibrium between Ce^3+^ and Ce^4+^, oxygen vacancies and highly mobile O^2−^ ions [11].

CeO_2_ NPs with improved quality and in relatively large amounts have been produced via a facile one-pot wet-chemical approach described in a previous work [11]. Therein, we were able to successfully define the synthesis parameters for single batches, which are readily scalable by means of continuous synthesis routes [4,12,13]. In the following we report the successful transfer of single-batch polyol syntheses of catalytically relevant CeO_2_ nanoparticles to the SFTR. 

**Figure 2:**
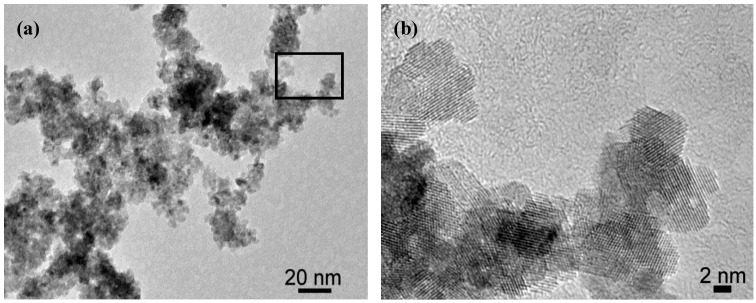
HR-TEM image of the as-prepared CeO_2_ nanopowders at (**a**) low and high (**b**) magnification. Figure 2b corresponds to the squared area of Figure 2a. Reprinted from [11] with permission.

**Figure 3:**
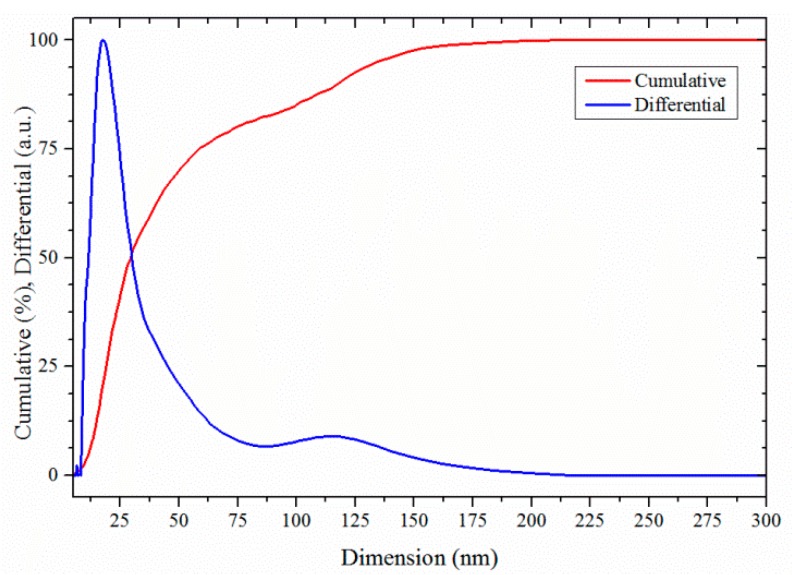
Particle size distribution (PSD) of the pure CeO_2_ sample after drying and re-dispersion in water, measured with an X-ray disc centrifuge. The blue line is the differential volumetric distribution and the red line is the cumulative volumetric distribution. Reprinted from [11] with permission.

As it can be seen from High Resolution TEM images (HRTEM, Figure 2), the as-prepared particles are loosely-packed and have an average crystallite size of 5 nm. The obtained samples were characterized by TGA, XRD, BET (Table 1) and an X-ray disc centrifuge to determine particle size distribution (PSD, Figure 3), before and after thermal treatment (500 °C for 1 h, heating rate: 3 °C/min).

**Table 1:** Summary of CeO_2_ nanoparticle properties before and after thermal treatment at 500 °C. The lattice parameter *a* and the crystallite sizes *d*_XRD_ were determined by high resolution synchrotron XRD and full pattern Rietveld refinement. The BET diameter, *d*_BET_, was calculated from specific surface area (SSA) measurements, assuming spherical particles and using the 5 points BET method. The TEM diameter, *d*_TEM_, was estimated from high resolution electron transmission microscopy (HR-TEM).

Sample	SSA (BET) (m^2^·g^−1^)	Lattice Parameter *a* (Å)	*d*_XRD_ (nm)	*d*_BET_ (nm)	*d*_TEM_ (nm)
as-prepared	169 (±5)	5.441_0_	4.7 (±1)	5.0 (±0.5)	5 (±2)
thermally treated (500 °C/1 h)	123 (±5)	5.413_9_	14 (±2)	6.9 (±0.5)	5–10

The specific surface area (SSA) decreased from 169 to 123 m^2^·g^−1^ upon thermal treatment, which is accompanied with a weight loss of 18%, which most likely is attributed to adsorbed water and the combustion of organic residues. With the upscaling of the polyol synthesis in the SFTR advantages from both approaches can be exploited: catalytically active nanoparticles with a relatively high SSA can be obtained in high quantities without using any additives, such as antiagglomerants or surfactants. With the settings described in the experimental section, a production rate of 2.75 g/h was realized, but even higher yields can theoretically be achieved.

The PSD analysis revealed the diameter of the main mode of the distribution is about 29 nm (Figure 3) and an agglomeration factor (*F_ag_* = *d*_v50%_/*d*_BET_) of about 6 can be deduced from the measured median volumetric diameter *d*_v50%_ and the BET diameter *d*_BET_.

The polyol-based preparation method described in this paper allows the facile one-pot synthesis of a relatively high amount of catalytically active material in a rather short time. By simple variations of the influxes for the stock solutions, we were able to realize a variety of single batch scenarios within shortest time and to easily obtain a library of products for parametric studies and screening purposes, which would be time-consuming without the SFTR. The synthesis has been extended for the continuous production of Ce_x_Zr_1−x_O_2_ solid solution as well as for the preparation of noble metal-doped samples. These new results will be subject of future publications.

### 2.2. Polyol Synthesis of Ni NPs

Transition metal NPs represent nowadays an attractive research area for many different applications. In catalysis, even if many works report the outstanding activity of NPs of Au, Pt, Pd, Ru and other expensive elements [14,15], the recent trend is to replace the expensive metal with a cheaper one, while still maintaining good catalytic activity [16,17]. One significant example can be found in the investigation of methanation reactions. This process is a crucial step in the production of synthetic natural gas: it offers the possibility to produce a high quality energy carrier without the consumption of natural resources and—when CO or CO_2_ are used as reagents—decrease the concentration of undesired gases [18]. In this framework, a nickel-based catalyst has been already demonstrated to be an attractive alternative to more costly ruthenium based catalyst [19]. Even if many studies deal with the possibility to tune the catalytic activity with the microscopic properties of the catalyst, a way to produce high amounts of a well reproducible and tailored catalyst were still missing. 

**Figure 4:**
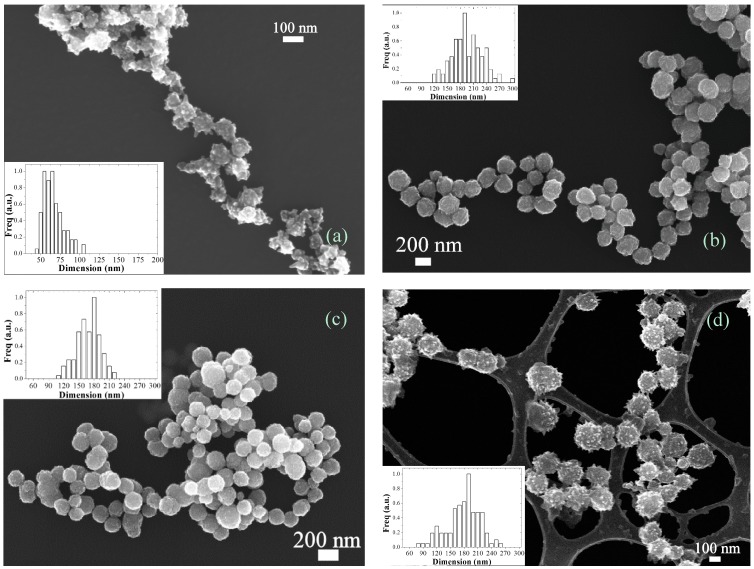
SEM images of samples S1 (**a**); S2 (**b**); S3 (**c**) and S4 (**d**) together with the correspondent diameter distribution.

For this reasons, we recently developed a robust synthesis method for the continuous production of tailored Ni nanoparticles based on the SFTR [20]. Thanks to its versatility, SFTR has proven itself to be an effective and reliable way to produce well-defined Ni NPs in the hundreds-gram-day scale. In particular, by tuning the parameters we produced NPs with dimensions ranging from 60 to 190 nm in diameter (Figure 4). Furthermore, the surface morphology of the particles can vary from spiky to raspberry-like by changing the relative amount of reducing agent. The modification of dimension/morphology of the particles also leads to different properties of the final product. For example, the magnetic properties (ferromagnetic or superparamagnetic) as well as the specific surface area (from 5 to 50 m^2^·g^−1^) can be precisely tuned (Table 2). The quality of the synthesis has been demonstrated for all the samples where phase purity was checked by high-resolution X-ray diffraction.

**Table 2:** Top: Some of the synthesis parameters applied in the experimental trial. Bottom: some product characteristics are reported. (d_SEM_: diameter determined from SEM images, SD: standard deviation, CV %: percentage coefficient of variance, d_XRD_: diameter from XRD data, SSA_BET_: specific surface area, BET method, FM: ferromagnetic, SPM: superparamagnetic).

**Synthesis Paramaters**										
	**Temp** **[K]**	**Concentration after Mixing [M]**					**Reactant Ratio**		**Aging Time [min]**	**Production** **[g·h^−1^]**
	**T_R_**	**C_Ni_**		**C_N2H2_**	**C_NaOH_**		**N_2_H_4_/Ni**	**N_2_H_4_/OH**	**t_A_**	
S1	343	0.05		1	0.075		20	13.3	2.9	2.1
S2	333	0.2		0.6	0.4		3	1.5	11.8	2.1
S3	333	0.05		0.15	0.1		3	1.5	5.3	2.1
S4	368	0.05		1	0.075		20	13.3	1.9	3.2
**Sample Properties**										
	***d*_SEM_** **(SD) [nm]**		**CV %**		***d*_XRD_** **[nm]**	**A_BET_ [m^2^·g^−1^]**		**Morph**	**Magnetic Behaviour**	
S1	60 (8)		13.3		10.90	51.7		Spiky	SPM	
S2	191 (28)		14.2		6.89	5.7		Raspberry	FM	
S3	167 (16)		11.5		6.85	9.9		Raspberry	FM	
S4	183 (21)		12.7		11.91	30.6		Spiky	FM	

The importance of the result is further highlighted when one considers the high number of applications where Ni NPs play a relevant role. In our work, we demonstrated that our particles show excellent activity as catalysts in the CO methanation reaction, retaining in a stable way a conversion higher than 83% after multiple cycles. In addition, the obtained product represents an ideal candidate for the preparation of advanced catalysts with improved stability and performances. At the moment, a superior catalyst with higher activity and thermal stability is investigated combining a metallic Ni core (produced in a continuous way thanks to the SFTR) with a porous silica shell. 

### 2.3. SFTR Polyol Synthesis of Ag NPs

**Figure 5:**
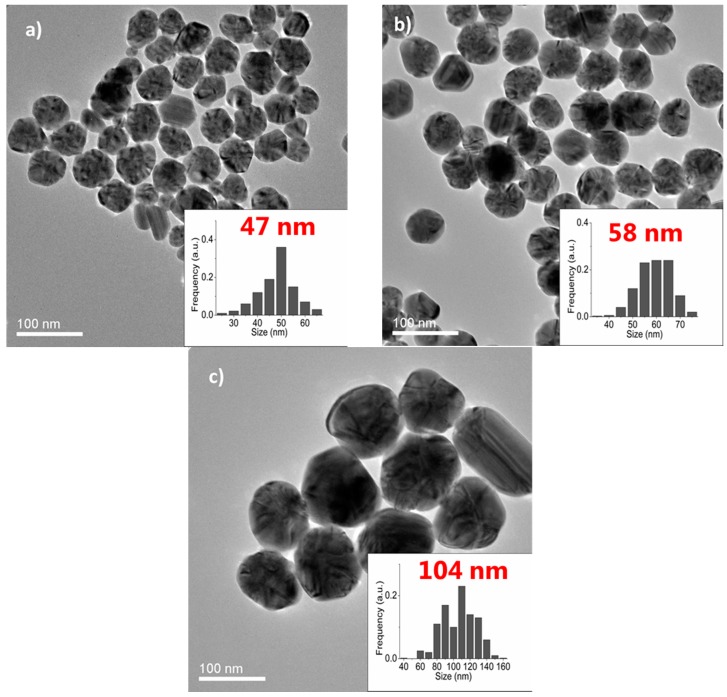
TEM micrographs of AgNPs obtained from the polyol synthesis performed in the SFTR. The average, TEM, particle sizes are: (**a**) 47 nm; (**b**) 58 nm and (**c**) 104 nm. Reprinted from [28] with permission.

AgNPs have attracted significant interest from various research groups due to their large scope of applications. Presently, AgNPs are employed in areas which include catalysis [21,22], biomedicine [23] optics [24] and electronics [25,26]. In general, silver nanostructures feature size-and shape-dependent properties, and therefore the preparation of AgNPs featuring a high degree of control over the shape, size and particle size distribution has become a key target [27]. Although various synthetic methods for the preparation of silver nanostructures exist, most of them suffer from drawbacks which include poor size- and shape-control, limited feasible particle sizes and toxic reagents/side products. Moreover, these reports rarely include information on the long-term sustainability of their continuous preparation method, and there have also been no thorough investigations on the preparation of NPs with various sizes. The syntheses of AgNPs with controlled particle size distribution had been conducted with the SFTR. Overall, the preparation of varying particle sizes (7–104 nm) was feasible through the modification of reaction conditions which consist of the reaction temperature and the molecular weight of the stabilizer (PVP). The continuous polyol synthesis could be performed for 4 h without significant fluctuations in the product quality which confirms the method’s ability for continuous particle production with enhanced control over the particle size and distribution [28]. The production rate was determined at ~2.3 g/h. A summary of the reaction conditions and the size parameters of the AgNPs is shown in Table 3, while Figure 5a–c illustrate the TEM micrographs of selected samples together with the particle size distribution. 

The large-scale preparation of AgNPs was inspired by their potential as a filler for high permittivity nanocomposites [29]. For this application, a relatively high amount of filler with controlled and reproducible particle size distribution was needed to produce, develop and test working devices. The SFTR products were able to fulfill these requirements. The properties of the nanocomposite devices are currently under testing [30,31,32].

**Table 3:** Summary of the reaction conditions and the size parameters of the prepared AgNPs.

Entry	Temperature (°C)	PVP *M*_w_ (g∙mol^−1^)	TEM size (nm)	DLS * size (nm)
S1	130	10,000	25 ± 4	20 ± 8
S2	140	10,000	47 ± 8	33 ± 12
S3	150	10,000	58 ± 7	56 ± 17
S4	130	40,000	7 ± 2	9 ± 2
S5	140	40,000	79 ± 15	107 ± 30
S6	150	40,000	104 ± 20	123 ± 32

* Dynamic Light Scattering.

### 2.4. SFTR Polyol Synthesis of β-tricalcium Phosphate (β-TCP), Ca_3_(PO_4_)_2_ Nanoplatelets

Calcium phosphate (CaP) ceramics have been successfully used as bone substitute materials due to their chemical similarity to the inorganic part of bone [33]. In particular, β-TCP is highly osteoconductive and allows for active cellular resorption [34]. Nevertheless, the brittle nature of ceramics prevents their use for load-bearing applications. A promising approach to solve this problem is to find inspiration in the highly organized nanostructures of natural materials [35,36]. For example, nacre owes its high toughness to a highly oriented composite structure of nanosized inorganic single crystals glued together by a thin organic layer. Recently, a CaP crystals synthesis method by precipitation in ethylene glycol was proposed by Tao *et al.* [37,38] and expanded by Galea *et al.* [39,40]. Thereby, highly uniform, non-agglomerated hexagonal β-TCP platelets with aspect ratios up to 15 and thicknesses below 200 nm could be produced. Current research is focusing on the preparation and mechanical analysis of CaP platelet-biopolymer composites. Moreover, platelets with low aspect ratios (controllable through experimental conditions) may be used to improve the injectability of bone cements and pastes with potential use for 3D printing applications. Nevertheless, the platelet synthesis, both in a batch and a small-diameter (1.6 mm) tubular reactor, is currently limited to less than 1 g/day, which underlines the importance of upscaling.

**Figure 6:**
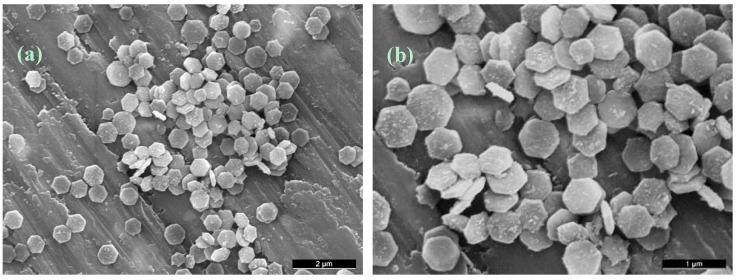
SEM images, (**a**) 10 kX and (**b**) 20 kX magnification, of β-TCP platelets produced in the SFTR reactor in ethylene glycol (EG) at 170 °C.

The SFTR allowed the production of hexagonal β-TCP platelets of 600 nm length and 100 nm thickness with a size dispersion of approximately 10% (Figure 6). The uniform and non-agglomerated nature of the platelets observed in the batch and small-size tubular reactor could thus be reproduced. Future work will examine the differences in platelet geometry as a function of experimental conditions, including temperature and pH, between the SFTR and the small-size tubular reactor. 

## 3. Experimental Section

### 3.1. CeO_2_ NPs

All chemicals were purchased from Sigma Aldrich (Buchs, Switzerland) and used as received. A modification of the procedure of Ksapabutr and co-workers was applied for CeO_2_ synthesis [13]. Two separate solutions of cerium(III) nitrate hexahydrate (Ce(NO_3_)_3_·6H_2_O, >99.0%) and sodium hydroxide (NaOH, >98%) were prepared in ethylene glycol (EG, puriss. p.a. >99.5%, c = 0.125 and 0.3 M, respectively) by dissolving each compound separately at 70 °C under magnetic stirring. After cooling down to RT, the two reactants were introduced into the reactor system by pumping with two HPLC pumps at a rate of 1.95 mL/min for the ceria precursor and 2.69 mL/min for the solution of NaOH in EG. After forming the reaction mixture in the micromixer, the micro-volumes were made in the segmenter by passing through dodecane (*n*-C_12_) at a speed of 24.8 mL/min. In the experimental trial, the temperature range was kept between 140 and 180 °C. The residence time of the micro-volumes within the tube in the thermostatic bath was 12 min. In a second tubular section, the segmented suspension was cooled down. The nanoceria suspension was then collected in the decantation funnel and separated from the *n*‑C_12_, which itself was recycled in the flow reactor as segmenting fluid. The particle suspensions were subjected to multiple cycles of washing in ethanol and then in acetone.

### 3.2. Ni NPs

Ni NPs were produced by the reduction of NiCl_2_·6H_2_O with hydrazine (N_2_H_4_·H_2_O), in the presence of NaOH in ethylene glycol (EG) as solvent. Two reagent solutions (sol A: 0.1 M NiCl_2_·6H_2_O in EG; sol B: 2 M N_2_H_4_·H_2_O, 0.15 M NaOH in EG) were previously prepared and connected to HPLC pumps. A third pump was connected to pure EG. The flowrates of the HPLC pumps, as well as the flowrate of the dodecane pump, were adjusted to reach the desired aging time at the reaction temperature. The reaction temperature was adjusted by tuning the temperature of the oil bath. The prepared particles were collected in a decantation funnel, spilled and washed several times with ethanol and acetone. The final powders were dried and stored in open air.

### 3.3. Ag NPs

All chemicals were purchased from Sigma Aldrich and used as received. Ethylene glycol (EG) was distilled prior use. A modified procedure from Xia and co-workers [31] was applied for AgNPs synthesis. Two separate solutions of AgNO_3_ (0.236 M) and PVP 10: *M*_w_ = 10,000 g·mol^−1^ or PVP 40: *M*_w_ = 40,000 g·mol^−1^ (2.16 M in terms of repeating units) were prepared. PVP and AgNO_3_ were dissolved at 70 °C under sonication and at room temperature under magnetic stirring, respectively. In order to protect the AgNO_3_ solution from sunlight, the container was shielded off by wrapping aluminum foil around it. The two reactants were introduced into the reactor system by pumping with two HPLC pumps at a rate of 1.5 mL/min. After forming the reaction mixture in the micromixer, the micro-volumes were made in the segmenter by passing through dodecane (*n*-C_12_) at a speed of 24.8 mL/min. In the experimental trial, the temperature range was kept between 130 and 150 °C. The residence time of the micro-volumes within the tube in the thermostatic bath was 12 min. The silver suspension was then collected in the decantation funnel and *n*-C_12_ separated. The reaction mixture was subjected to multiple cycles of washing with an ethanol/acetone mixture followed by redispersing in ethanol for further use and analysis.

### 3.4. Ca_3_(PO_4_)_2_ Nanoplatelets

Calcium and phosphate precursor solutions with a Ca:P molar ratio of 3:2 were prepared as follows: For the calcium solution, 1.43 g CaCl_2_∙2H_2_O (CaCl_2_·2H_2_O, Merck, Schaffhausen, Switzerland) were dissolved in 500 mL ethylene glycol (EG, VWR, Dietikon, Switzerland). For the phosphate solution, 19.4 mL of a 0.33 M Na_2_HPO_4_ (Na_2_HPO_4_·2H_2_O, Fluka, Buchs, Switzerland) solution and 1.71 mL of a 1.3 M NaOH (NaOH, Fluka) solution were added to 500 mL ethylene glycol. The two reactants were introduced into the reactor system by pumping with two HPLC pumps at a rate of 3.0 mL/min and the reaction time was fixed to 10 min. The resulting platelet suspension was cooled at room temperature and centrifuged in 50 mL tubes at 4000 rpm for 30 min to extract the solid phase. The crystals were then re-dispersed and centrifuged in ethanol, in demineralized water and again in ethanol for 10 to 15 min each. Subsequently, the precipitate was re-dispersed in ethanol and an aliquot was spread over a scanning electron microscopy (SEM) aluminum sample holder and dried in air to allow for platinum sputter coating and image acquisition.

## 4. Overall Conclusions 

In this paper we summarize the synthesis procedures and main results for four simple components as examples of the extended ability of the SFTR concept for the production of NPs in organic solvents at temperatures >100 °C compared to standard aqueous systems. This polyol route allows the preparation of material with outstanding properties thanks to a series of positive effects:

(i) Due to the polarity of the polyols (ε > 30), inorganic precursor salts are often very soluble [41];
(ii) It is possible to apply relatively high temperatures at ambient pressure simplifying the equipment used for the synthesis;
(iii) Because of the high temperature, well-crystallized materials can be prepared [42,43,44]. High crystallinity is often a mandatory material property;
(iv) The chelation effect of the solid by polyol limits the particle growth and prevents particles agglomeration [43,45];
(v) Since the polyols are normally low-weight molecules, they can be removed from the particle surface under certain experimental conditions [43,45];
(vi) The synthesis protocols can be extended to water free systems;
(vii) Polyol ensure a reducing chemical environment, allowing the synthesis of materials sensitive to oxidation [12];
(viii) Density and viscosity of ethylene glycol are fully compatible with SFTR plants designed for water based synthesis;
(ix) The organic solvent may be recovered by distillation and re-used;
(x) The SFTR concept can be easily applied for the synthesis of organic compounds and pharmaceutical products.

In our opinion, the combination of SFTR and polyols synthesis will open a vast portfolio of synthesis options for the production of high quality NPs in amounts adequate for the realization of and development of innovative materials. Currently, new systems are under investigation such as the continuous synthesis of Metal Organic Frameworks (MOFs) and precious-metal-doped mixed-oxide compounds (e.g., Pt/Pd doped Ce_(x)_Zr_(1−x)_O_2_) for catalytic applications. A possible future improvement of the SFTR could be devoted to the realization of new a configuration operating under hydrothermal conditions up to 250 °C.
